# MicroRNAs Overexpressed in Growth-Restricted Rat Skeletal Muscles Regulate the Glucose Transport in Cell Culture Targeting Central TGF-β Factor SMAD4

**DOI:** 10.1371/journal.pone.0034596

**Published:** 2012-04-10

**Authors:** Santanu Raychaudhuri

**Affiliations:** Department of Pediatrics, Department of Microbiology, Immunology and Molecular Genetics, David Geffen School of Medicine, University of California Los Angeles, Los Angeles, California, United States of America; University of Maryland School of Medicine, United States of America

## Abstract

The micro-array profiling of micro-RNA has been performed in rat skeletal muscle tissues, isolated from male adult offspring of intrauterine plus postnatal growth restricted model (IPGR). Apparently, the GLUT4 mRNA expression in male sk. muscle was found to be unaltered in contrast to females. The over-expression of miR-29a and miR-23a in the experimental group of SMSP (Starved Mother Starved Pups) have been found to regulate the glucose transport activity with respect to their control counterparts CMCP (Control Mother Control Pups) as confirmed in rat L6 myoblast-myocyte cell culture system. The *ex-vivo* experimentation demonstrates an aberration in insulin signaling pathway in male sk. muscle that leads to the localization of the membrane-bound Glut4 protein. We have identified through a series of experiments one important protein factor SMAD4, a co-SMAD critical to the TGF-beta signaling pathway. This factor is targeted by miR-29a, as identified in an *in vitro* reporter-assay system in cell-culture experiment. The other micro-RNA, miR-23a, targets SMAD4 indirectly that seems to be critical in regulating insulin-dependent glucose transport activity. MicroRNA mimics, inhibitors and siRNA studies indicate the role of SMAD4 as inhibitory for glucose transport activities in normal physiological condition. The data demonstrate for the first time a critical function of microRNAs in fine-tuning the regulation of glucose transport in skeletal muscle. Chronic starved conditions (IPGR) in sk. muscle up-regulates microRNA changing the target protein expression patterns, such as SMAD4, to alter the glucose transport pathways for the survival. The innovative outcome of this paper identifies a critical pathway (TGF-beta) that may act negatively for the mammalian glucose transport machinery.

## Introduction

Tissue-specific, developmental and stress-induced expression patterns of a group of microRNAs regulate essential functions in biological systems [1]–[3]. These small RNA elements are powerful post-transcriptional regulators in altering gene expression to orchestrate the normalization of physiological activities under stress conditions. Thus, it is likely that aberrant expression of microRNA leads to disease conditions including carcinogenesis and metabolic syndromes. The glucose transporters in peripheral tissues, such as skeletal muscles, are pivotal in regulating glucose transport activity and thus balance glucose homeostasis in the blood. In response to insulin, ischemia and exercise, GLUT4 molecules translocate into the plasma membrane and orchestrate facilitative glucose transport into the cells [4]. Insulin-dependent translocation of GLUT4 vesicles into the plasma membrane is the major mechanism by which glucose uptake into the sk. muscles and cardiac muscle tissues can be often regulated [5], [6]. Aberration in skeletal muscle glucose transport pathway can cause metabolic diseases including insulin resistance and diabetes [7]–[12]. Groups of tissue-specific (e.g., miR-1, miR-206, miR-208) and non-tissue-specific (e.g., miR-29a, miR-23a) microRNAs have been found to control skeletal muscle development in growth and differentiation [13]–[19]. The tissue-specific microRNAs can regulate glucose homeostasis and the pathophysiology of metabolic disease [20]–[22]. The expression of GLUT4, both transcriptional and translational, and its membrane trafficking from cytoplasmic vesicles upon insulin signaling, is critical in glucose transport activity of sk. muscles in both normal physiological and metabolic disease conditions [23]–[26]. Intrauterine growth restriction (IUGR) model mediated by various causes (e.g., semi-calorie food restriction, protein restriction, hypoxic condition in rodents) has been shown to alter the insulin signaling in offspring, leading to the development of insulin resistance in the sk. muscles [27]–[31]. The transcriptional changes of GLUT4 expression in female rat under these conditions has been attributed to the epigenetic changes including histone modifications, histone deacetylation (HDAC recruitment) and other associated changes in key enzymes of this process [32]. The histone code modifications were also been inferred in IGF1 transcription of IUGR rat offspring in programmed obesity [33]. The inappropriate nutrition in the early intrauterine growth phase can have a deleterious effect on the adult life, such as metabolic syndrome [34]. All of these observations raise the possibility of trans-generational epigenetic changes that may have occurred in the intrauterine environment upon nutritional interruptions/aberrations, thus the offspring get susceptible to the development of phenotype leading to metabolic disorders. While investigating the GLUT4 status of the male counterpart skeletal muscle, no change was observed in total GLUT4 expression overall in comparison to the female counterpart in IPGR offspring. This differential, gender-specific transcriptional control of GLUT4 under this food restriction protocol led me to investigate the global microRNA gene expression pattern in male sk. muscles and thus the involvement of these small regulatory genetic elements in the glucose transport process. MiR-223 and miR-133 regulate the expression of glucose transporter 4 in cardiomyocytes either by directly targeting GLUT4 3′UTR or indirectly targeting other protein-coding mRNA, e.g., KLF15 [35], [36]. MiR-223 up-regulation in cardiomyocytes causes the phosphatidylinositol-3-kinase (PI-3K) independent increase of glucose transport activity [36]. The miR-29 group of microRNAs was found to be up-regulated in muscle and fat tissues of Goto–Kakizaki rats, a non-obese rat model of diabetes mellitus (T2DM). The over-expression of miR-29 in 3T3-L1 adipocytes inhibited insulin-stimulated glucose uptake [37].

The cutting edge LNA-based microarray of microRNA in male skeletal muscle revealed a number of microRNAs were up- and down-regulated in the experimental SMSP (Starved Mother Starved Pup; Semi-calorie food-restriction model) animal offspring compare to CMCP. I began investigating the functional significance of these microRNAs in regulating the glucose transport activity in a rat cell culture system. MicroRNA 29a and 23a proved to be significant in fine-tuning glucose transport activity in an insulin-independent and dependent manner, respectively. Finally, an effort was made to discover some of the regulatory components of this essential functional glucose transport activity in sk. muscle targeted by specific microRNA(s). I revealed the observation that SMAD4 (mothers against decapentaplegic homolog 4), a co-SMAD of the TGF-β pathway, may play a significant role in linking the core metabolic pathway with skeletal muscle growth and differentiation.

## Materials and Methods

### Antibodies, LNA-based Pre-miR(s), anti-miR(s) inhibitors, siRNA(s)

The antibodies used for Western Blot analysis are Glut4 (Abcam, ab654), Glut3 (made in Devaskar's lab, UCLA), Glut1 (Abcam, ab15309), IGFR (Cell Signaling, 111A9), EGFR, (Santacruz, sc 03, 1005), Glucose-6-P dehydrogenase (Abcam, ab34436), Protein Kinase C-zeta (Cell signaling, ab51157), P-PKC-zeta (Abcam, ab76129), Syntaxin 4 (Abcam, ab57841), Vamp2 (Abcam, ab3347), Vimentin (Santacruz, sc73259, Actin (Santacruz, sc-1615), Nucleolin (Santacruz, sc13057), Lamin A (Santacruz, sc-20680), MEF2C (Abcam, ab65252), HDAC4 (Cell Signaling, #2072), HAND2 (Abcam, ab56590), Tropomyosin C (Santacruz, sc73225), DnaJB1 (Santacruz, sc-1800) and SMAD4 (Abcam, ab1341). *Precursor microRNA mimics-* Mimics of endogenous precursor micro-RNA synthetic molecule for rat, namely, Pre-miR-1, Pre-miR-23a, Pre-miR-23b and Pre-miR-29a were purchased from Ambion Inc. for gain-of-function studies in the rat cell-culture system. Pre-miR™ miRNA Precursor (AM17110) is a double-stranded RNA oligonucleotide designed to serve as a negative control for experiments involving Pre-miR miRNA precursors. *Anti-miR(s) inhibitors* - miRCURY LNA™ microRNA Inhibitors are pre-designed microRNA inhibitors with a novel design by Exiqon Inc. The micro-RNA inhibitors have been optimized with respect to potency and stability against each of the ready-made LNA-based anti-sense oligonucleotides. *siRNAs* have been used from Dharmacon SMARTpool system. DharmaFECT transfection reagent was used for siRNA experiment.

### Animal

Sprague-Dawley rats (Charles River Breeding Laboratories, Hollister, CA) were housed separately in cages, exposed to 12∶12-hour light-dark cycles at 21–23°C, and allowed *ad libitum* access to standard rat chow. Animal care was approved by the Animal Research Committee at UCLA in accordance with the guidelines from the NIH, Bethesda, Maryland. The skeletal muscle tissues were given to the author for this *in vitro* study. The author did not raise the animals for *in vivo* studies. The maternal semi-nutrient restriction model and the postnatal animal maintenance were detailed in Shilpa AO, et al., 2006 [38]. The experimental group (SMSP or IPGR) was generated after cross-fostering between intrauterine semi-nutrient (50% food intake, e11–e21 gestation) restricted pups (SP) and semi-nutrient (50% food intake, d1–d21) restricted mothers (SM). The control animals were made by the same way using control (ad-libitum food 22 g/day, e11–e21) pups (CP) and control mothers (CM). 450 days of postnatal adult male rats were used for this study.

### RNA isolation, micro-RNA enrichment, RNA quality

Total RNA was isolated from rat skeletal muscles using Invitrogen Trizol reagent. The muscle tissues were cut into pieces and frozen in liquid nitrogen, then ground into powder in mortar and pestles and stored at −80°C. For enriched fraction of micro-RNA isolation quantitatively from tissues and cells, the mir*Vana* miRNA isolation kit of Ambion was used. RNA quality was measured by mass spectrometry and agarose-gel electrophoresis by examination of the band integrity of ribosomal RNA(s).

### Microarray analysis

The Exiqon (Vedbaek, Denmark) miRCURY LNA microRNA arrays and service to process the samples were used. A mixture of equal amounts of total RNA from the sk. muscle of control (CMCP) and the experimental (SMSP/IPGR) were used as the reference pool. A total of 2 µg RNA from each sample was then labeled with the Hy5™ fluorescent label and the reference pool labeled with Hy3™ using the miRCURY™ LNA Array labeling kit (Exiqon). The labeled samples and reference pool were then mixed pair-wise and hybridized to the miRCURY™ LNA array containing capture probes targeting all rat miRNAs listed in the miRBASE version 8.1 (Exiqon). In addition, the array contained capture probes for another 10 spike-in control microRNAs (Exiqon), which can be used for further calibration or control of the profiling experiment. After hybridization, the slides were scanned, quantified signals and normalized by Exiqon using the global Lowess (Locally Weighted Scatterplot Smoothing) regression algorithm.

### RT-PCR analysis

The mir*Vana* miRNA isolation kit along with the mir*Vana* qRT-PCR primer sets (including reverse primers for RT reactions) of Ambion were used for semi-quantitative micro-RNA analysis. The first step for this protocol was RT reactions of microRNA enriched RNA molecules followed by the synthesis of amplified PCR reactions instead of qPCR analysis. The decision was made to follow the regular controlled PCR reactions with optimum cycles determined in separate reaction sets using the specific RT primer of Ambion and the micro-RNA specific primers designed by the same company.

### Northern Blot Analysis

The total RNA sample (10 µg) was run on a denaturing 12% polyacrylamide gel containing 8 M urea and the fractionated RNA samples were then transferred to Nytran-N (Amersham Bioscience) membranes by capillary method and fixed by UV cross-linking according to the manufacturer's instructions. For radio-labeling of the miRCURY™ probe, 20 pmole probe was used for the end-labeling reaction combined with gamma-32P-ATP and T4 polynucleoitde kinase according to standard protocol. The membrane was pre-hybridized in small RNA hybridization buffer. (50% formamide, 0.5% SDS, 5×SSC, 5×Denhardt's solution, and 20 µg/ml sheared denatured salmon sperm DNA). The membrane was hybridized in the same solution at 45°C overnight after addition of the labeled miRCURY™ probe (Exiqon) boiled for one minute. The membranes were washed at low stringency in 2×SSC, 0.1% SDS at room temperature twice for five minutes followed by high stringency washes @ 0.1 SSC, 0.1% SDS at 65°C twice for five minutes. The membranes were then exposed in X-ray film and incubated for 48 hours followed by visualization through autoradiography.

### Cloning, transfection and luciferase assay

The vector pMIR-REPORT (Luciferase-based) is designed for the cloning and testing of putative miRNA binding sites (Ambion Inc.). This plasmid can be transfected into mammalian cells to evaluate either endogenous miRNA expression, or to evaluate up- or down-regulation resulting from the transfection of Pre-miR™ miRNA and Anti-miR™ miRNA molecules. The 3′UTR of the SMAD4 gene was cloned into the Hind III and SpeI sites of reporter MCS. The luciferase expression is driven by the CMV promoter *in vivo*. The addition of the target gene 3′UTR in the downstream luciferase gene (as the 3′UTR) alters its expression pattern in RNA translational levels. Upon transfection in the cell culture system along with or without Pre-miRs and Anti-miRs for 48–60 hrs, the luciferase activity and the protein concentration were measured in a Luminometer using Promega luciferase assay reagent and Biorad reagent assay, respectively, according to the manufacturer's protocols. The negative control Pre-miR, Negative Control #1, miRNA of Ambion is a non-targeting sequence that bears no homology to the sequences of human, mouse, or rat transcripts. 25 nM final concentrations of both Precursors mimic micro-RNA and anti-miR miRNA inhibitors were used in this study. Transfection of muscle cells with micro-RNA were done using either siPORT NeoFX transfection reagent from Ambion, with siRNA done using Lipofectamine RNAiMax, and with plasmid DNA done using Lipofectamine 2000 from Invitrogen.

### Cell culture analysis

Mammalian skeletal muscle cell-lines (bought from ATCC) of rat origin L6 myoblasts and H9c2 cardiomyocytes (embryonic heart tissue, myoblast morphology) were used in this study. L6 myoblast cells were maintained according to the ATCC protocol using DMEM with 4 mM L-glutamine adjusted to 0.15% bicarbonate and 0.45% glucose in addition to 10% FBS. The cells were maintained in low confluency with 3 passages every week with change of medium every 2–3 days unless stated otherwise for any particular experiment. All the experiments involving the L6 cell-line were performed within 10 passages from initial thawing. Once the cells were received from ATCC, cells were cultured for 2–3 passages, then aliquoted and frozen in liquid nitrogen. For myocyte-myotube formation of the skeletal muscle cells, serum deprivation for (mostly 2%) 8–12 hrs was manifested. For transfection experiments, cells were seeded 2 days early with 30–40% confluence. By the end of 48 hrs, the plates were populated with 70% confluence.

### Glucose transport assay

The basic protocol for glucose transport assay in rat L6 cell culture system is taken from Klip et al., 1984. L6 cells were grown in DMEM with regular 10% FCS and 4.5 mM glucose until reaching 80% confluence followed by changing medium with α-MEM with 2% FCS to saturation in 12 well plates. The differentiation into myocyte-myotube formation was tracked by looking at the multinucleated cells under a microscope. Cells were washed by 2× α-MEM/0.1% BSA (pH 7.4) followed by serum-free step-down for 6 hours in the same medium in a 5% CO_2_ incubator. Cells were washed in 2× KRH/0.1% BSA followed by glucose-free step down with or w/o insulin for 45 minutes (50 nM) in 0.45 ml KRH/0.1% BSA at 37°C. Plates were washed three times quickly with reaction buffer (150 mM NaCl, 5 mM KCl, 1.2 mM MgSO_4_, 1.2 mM CaCl_2_, 2.5 mM NaH_2_PO_4_, 10 mM Hepes pH 7.4, 0.1% BSA). 0.5 ml of this buffer was added to each well and the transport reaction was started with the addition of radioactive (0.5 µCi ^3^H-OMe-Glucose and/or ^14^C-2-deoxy glucose) substrate of final concentration of 0.15 mM. Cytochalasin B (Sigma), 10 µM, and insulin (Sigma, bovine pancreas), 50 nM, were also used during the transport reaction if permitted for the experiments. The transport reaction was continued for 6 minutes (linear phase under the conditions, data not shown) terminated by vacuum suctioning off the reaction solutions immediately followed by cold wash with PBS, three times. The cells were dissolved in 0.1 N NaOH, 0.5 ml followed by neutralization with 1 N HCl. A fraction of the cell extract was used to measure both radioactivity in the scintillation counter and protein concentration by Bradford reagent.

### Tissue fractionation for Western Blot analysis

The compartmental cell fractionation kit of Chemicon Inc./Millipore was used to isolate the proteins from rat skeletal muscles into cytosolic, nuclear, membrane and cytoskeletal fractions. The protocol was standardized by SDS-PAGE and Western Blot analysis using various protein markers for compartmental proteins. GAPDH, Histone, EGFR and Vimentin were used for cytosolic, nuclear, membrane and cytoskeletal proteins. The SKM samples were pulverized under liquid nitrogen and stored at −80°C followed by power homogenization for four times. Equal amount of proteins were loaded in SDS-PAGE from control (CMCP) and experimental (SMSP) samples, then run and transferred onto PDVF (Invitrogen Inc.) membranes for Western Blot analysis. Primary antibodies described in the antibody section were used for the blots according to the manufacturer's protocol using either 5% milk, 3% BSA, or just PBS-Tween depending on the quality and nature of the antibodies. Anti-rabbit, anti-mouse or anti-goat horse-radish peroxidase (HRP) tagged secondary antibodies were used against the appropriate primary antibodies for luminol-based ECL/ECL-Plus detection.

### Statistical analysis

Student's T-test was always performed between two sets of data, for the control versus each experimental micro-RNA treated. The intention was to see the significance between these two groups, rather than among the groups performed in some experiments. The criteria for T-test significance was based on the two-tailed distribution and for paired or equal variance type.

## Results and Discussion

### Glut4 mRNA expression level remains unaltered in male IPGR rat SKM

**Figure 1:**
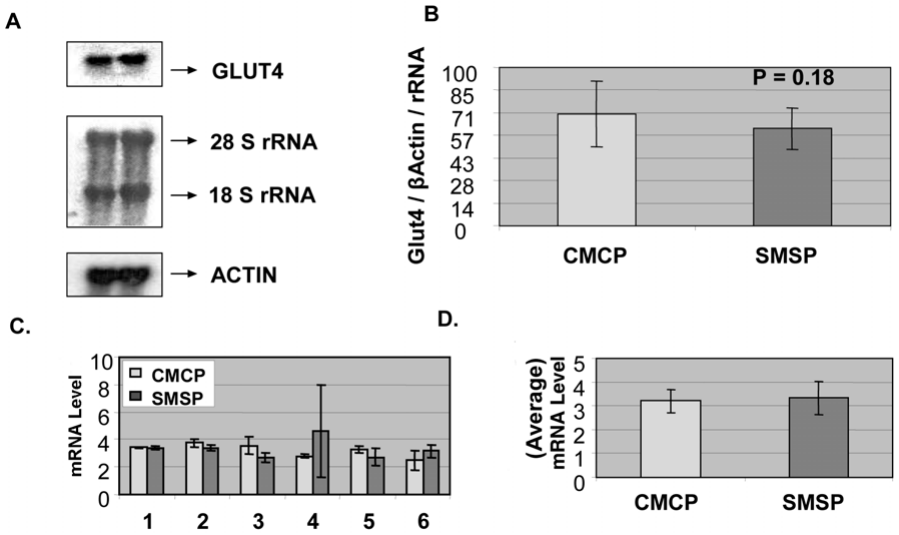
Glut4 mRNA expression level does not alter with the IPGR semi-calorie food restricted male rat skeletal muscles. A. Representative Northern blot analysis using total RNA isolated from six male rat SKM samples (450 days-IPGR) of both control (CMCP) and food restricted animals (SMSP). GLUT4 and β-Actin specific probes were used in hybridization. Ethidium bromide stained 28S and 18S ribosomal RNA is used as loading control. β-Actin is used as internal control. B. Intensity of the band was quantitated and expressed as percentages. C. Real-time PCR has been performed using the same RNA samples (1–6) of CMCP and SMSP male rat SKM using GLUT4 specific TaqMan probe and primers using GAPDH, Glyceraldehyde-3-phosphate dehydrogenase, as internal control and D. Average of six samples.

The predominant isoform of glucose transporter 4 is essential in functioning as a carrier of glucose molecules across the cell membrane *in vivo*, specifically in skeletal muscles and adipose tissues of mammalian origin [39], [40]. We have found the expression of this pivotal membrane-spanning protein is diminished in female rat SKM under the experimental calorie-restrictive starved conditions both *in vivo* and *in vitro* cell-culture conditions [32]. Major transcriptional networks in Glut4 expression have been studied in this system and a number of epigenetic factors were found to be responsible for this diminution of Glut4 expression level. Having found the significance for this restricted glucose transport activity in female rat sk. muscles in the development of insulin resistance in future progeny of the starved pregnant mother, I became interested to find out the trend of Glut4 expression in the male rat counterpart for possible detailed study from the epigenetic point of view. No changes were detectable in Glut4 mRNA expression in the male SMSP group of the study compared to CMCP. In Fig. 1, the northern Blot (a, b) and real-time RT-PCR (c, d) has been performed using total RNA of the SKM samples (n = 6). The relative intensity of Glut4 per unit of β-Actin and rRNA species is expressed for two groups and no statistically significant result was found between the groups (p = 0.18). This observation was verified by real-time PCR using the same total RNA and led to the same conclusion. This contrasting result between the gender samples in the IPGR model left several questions unanswered regarding the regulation of gene expressions in this regard. The major question that arose was how the male SKM counterpart was resistant in Glut4 expression status and what phenotypic differences were possible under the experimental conditions compared to female rats. To investigate this gender-specific difference in gene expression, the focus was placed on the translational control of mRNA that might be important in regulating GLUT4 mRNA translation given the importance on the regulation of initiation of translation after stress [41]. As microRNA controls the translational initiation by partial binding to the 3′UTR of many mRNAs, and the aberration of microRNA expression has been considered to be significant in regulating the epigenetic status of the muscles tissues [42]–[45], a bioinformatics analysis was made regarding the possible putative binding sites for muscle-specific or any putative microRNA that may regulate the GLUT4 expression in possible alternative mechanistic routes. Unfortunately, there is no microRNA known or found in the bioinformatics searches (e.g., Sloan-Kettering microRNA site and mIRANDA) that could potentially bind the Glut4 mRNA directly on its 3′UTR sequences at that time of investigation. The alternative possibility of affecting Glut4 expression and thus the glucose transport activity in SKM is through an indirect mechanistic process of altering the signaling associated with GLUT4 translocation in the membrane from the cytosolic reservoirs [46]. Any targeting by microRNAs would reveal and possibly explain the differential status of expression patterns in a gender specific manner. This, thus, led to the investigation of global microRNA expression patterns in the experimental SMSP samples compared to the control male rat muscles. This would reveal the miRNA-specific targeting of muscle glucose transport activity, if there is any.

### Identifications of microRNA(s) that are either over or underexpressed in the experimental SMSP skeletal muscle samples

**Figure 2:**
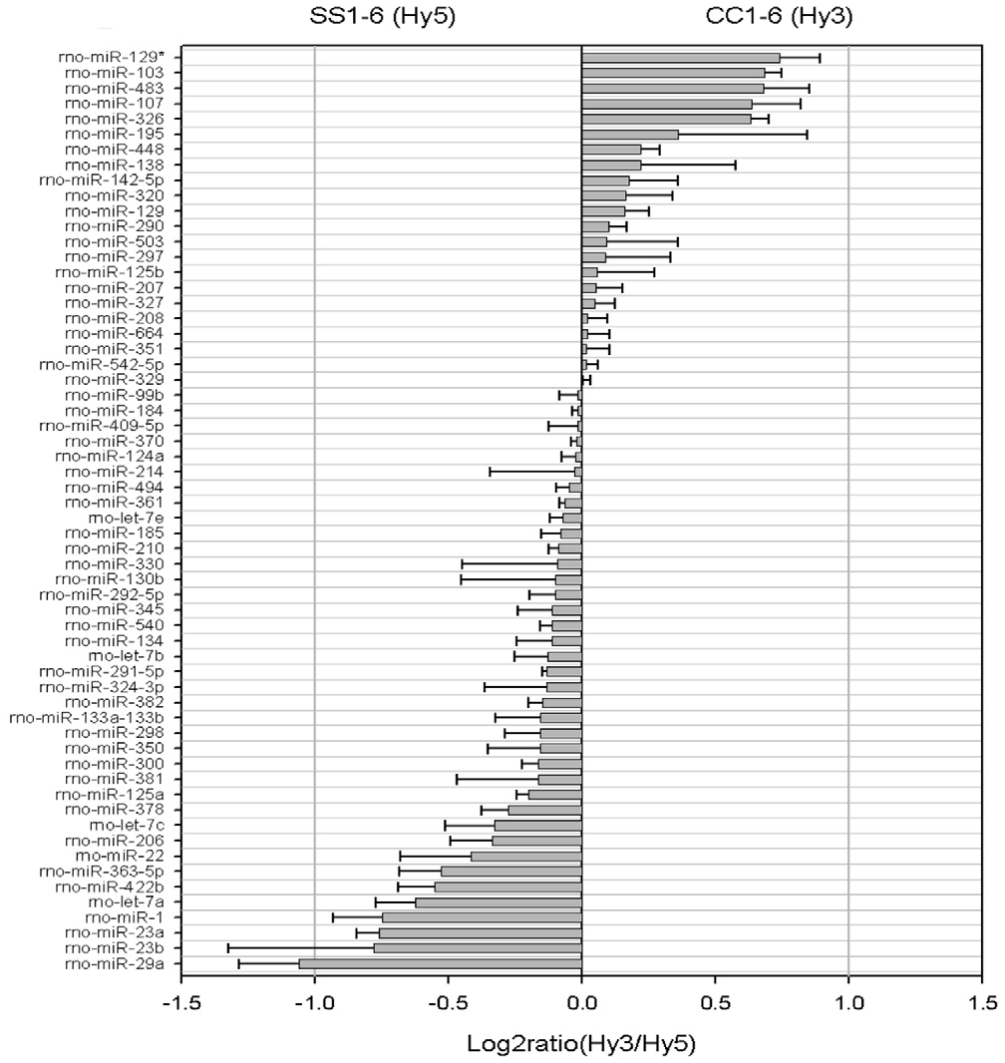
Over-expression of some microRNAs is observed in IPGR SKM samples over the control animals. Differential expression profile of sixty micro-RNAs that bind to the Chips in LNA-based microarray analysis (Exiqon miRNA-vol 8.1) of male rat SKM micro-RNA enriched samples isolated from CMCP (CC1-6) and SMSP (SS1-6) animals. CC1-6 samples are labeled with Hy3 and SS1-6 by Hy5 dyes. Log2ratio of Hy3 by Hy5 is plotted against every bound microRNAs. Sample size was N = 3 and 10 different spike-in controls have been used for the experiment as internal control.

**Figure 3:**
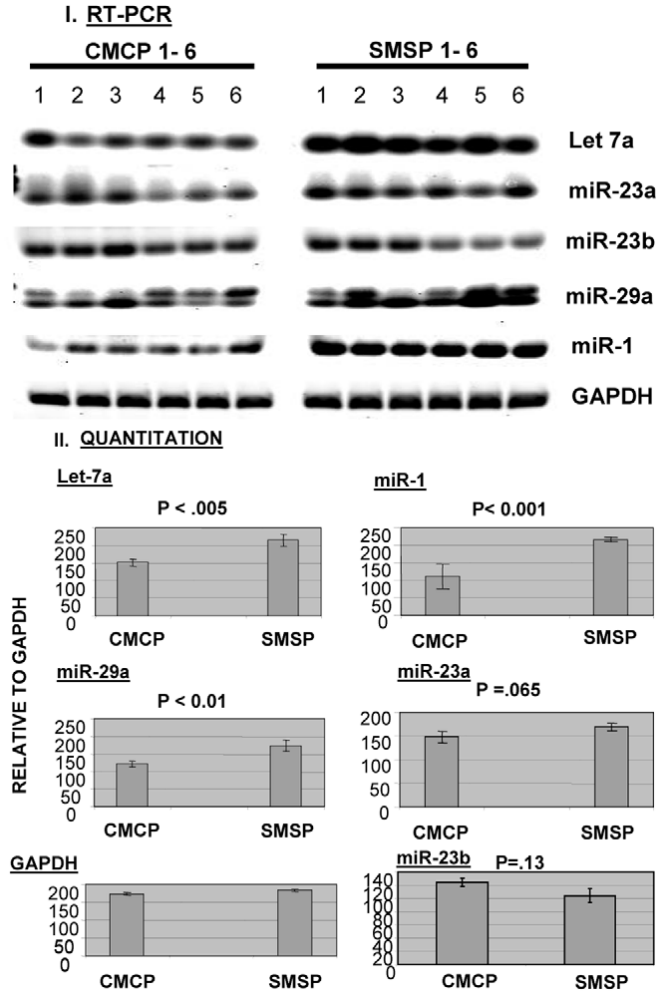
Semi-quantitative RT-PCR analysis of microRNAs in male SKM samples. **I.** The total RNA (1 µg) has been used to make cDNA using Ambion mirVana isolation kit. Equal amount of cDNA has been used in separate microRNA-specific PCR reactions. CMCP (1-6) and SMSP (1-6) correspond to six samples of control and starved SKM. **II.** Image J analyses of the intensities of all bands were plotted as graphs for each microRNAs. T-test was done as a measure of statistical analysis. GAPDH was used as an internal control.

**Figure 4:**
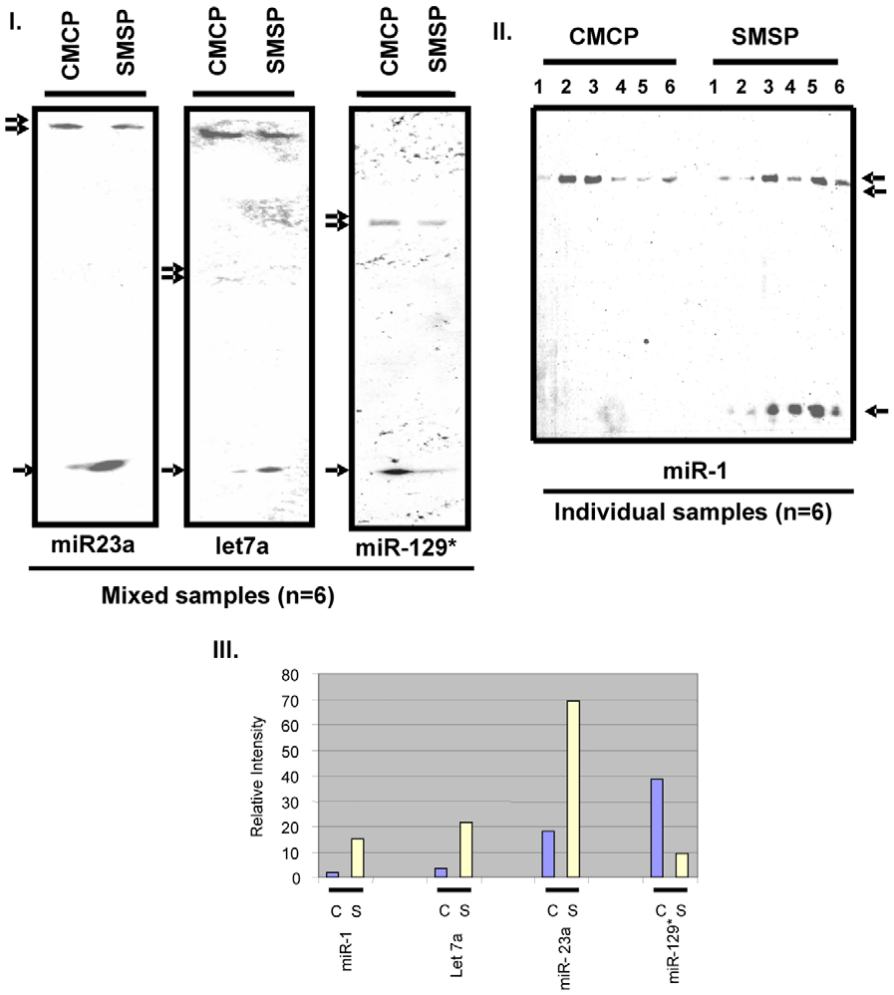
Analyses of mature microRNAs of interest found in both micro-array and RT-PCR analysis. Denaturing Urea-Acrylamide gel electrophoresis of total RNA (10 µg) have been run and subjected to Northern Blot analysis using P^32^-labelled LNA-based microRNA probes (Exiqon) either as mixed samples of 6 SKM RNA (**I**) or separately for 6 samples for miR-1 (**II**). The single arrow stands for the mature microRNA of appropriate size and the double arrow signifies the precursor forms, 60–100 bases. SMSP is the experimental against CMCP as control animal samples. **III**. Relative intensities of the bands in I and II.

LNA-based miRCURY (Vol. 8.1) microRNA array technology by Exiqon Inc. was utilized to find the group of microRNAs that might be over-expressed in the SMSP group relative to the control CMCP tissues. This locked-nucleic acid based microarray technology offers the most sensitive and specific hybridization with the sample RNA preparations using the Tm normalized capture probes and by spike-in control microRNA probes incorporated within the reaction mixtures. The CMCP and SMSP total RNA from the equimolar mixtures of n = 6 RNA samples prepared from Ambion's miR-Vana isolation kit was verified for their integrity by mass-spectrometry (figure not shown) and then labeled by Hy3 and Hy5 dye respectively. The experiment was performed in triplicate and regression analysis was done for reproducibility (R^2^ = 0.99). Ten different spike-in controls were used for assessment of the data quality. Spot morphologies were obtained and intensities were scanned using the Exiqon software program. The log2 ratio of Hy3 to Hy5 was plotted for all 61 microRNAs that bind the template harboring rat 261 microRNAs in the hybridization process (Fig. 2). The over-expression of miR-29a, 23b, 23a, 1 and let7a in SMSP (1–6):Hy5 and miR-129*, 103, 483, 107 and 326 in CMCP (1–6):Hy3 have been observed. Only two-fold or nearly two-fold over-expression has been observed in some cases, mostly in miR-29a for SMSP and miR-129* for CMCP. An endpoint RT-PCR miRNA detection kit using Ambion miRVana qPCR was used. Figure 3 shows the 22 cycles (mid log of exponential phase, data not shown) of end products in RT-PCR reactions for let7a, miR-23a, miR-23b, miR-1 along with GAPDH as an internal control. Distinctively, miR-1, let7a and miR-29a expressions were much higher in SMSP experimental samples (n = 6) than in control CMCP rat muscles. Density scanning of the bands revealed a statistically significant difference (p<0.05) of ∼1.5–2.0 fold increase of the microRNA expressions as found in the microarray analysis (Fig. 3. I). MiR-23a seemed to have higher expression in experimental tissues but not statistically significant levels (p = 0.065), whereas miR-23b expression was not altered like in internal control GAPDH (p = 0.13; Fig. 3. II). This small microRNA miR-29b is serving also as an internal control because this regulatory molecule turned out to be not significant. In order to see the effective mature microRNA molecules, denaturing polyacrylamide gel electrophoresis was conducted followed by Northern Blotting using ^32^P-labeled miR-23a, miR-129*, miR-1 and let7a specific LNA Probes. MiR-129* is under-expressed in the experimental samples compared to the control (Fig. 4, panel I). This was included in the gel electrophoresis in order to verify the authenticity of the assay. Mature miR-23a was found to be over-expressed as revealed in the LNA-based microarray (Fig. 4, panel 1). Northern Blotting also revealed the precursor molecules (70–100 nucleotides) for individual microRNAs. Surprisingly, in miR-1 Northern Blot, almost no detectable mature miR-1 was found in control CMCP compare to SMSP, whereas the level of precursor forms remained unaltered (Fig. 4, panel II). This may suggest an unidentified mechanism becomes activated in order to generate the mature miR-1 molecules in the experimental starved adult rat male SKM. Fig. 4 Panel III shows the relative intensities of these mature microRNAs with approximately 3–4 fold differences between the experimental and control groups.

### Membrane Glut4 localization is up-regulated in starved SKM while the Insulin-signaling pathway becomes positively regulated: Identification of few possible microRNA targets that may play important role in glucose transport in SKM

**Figure 5:**
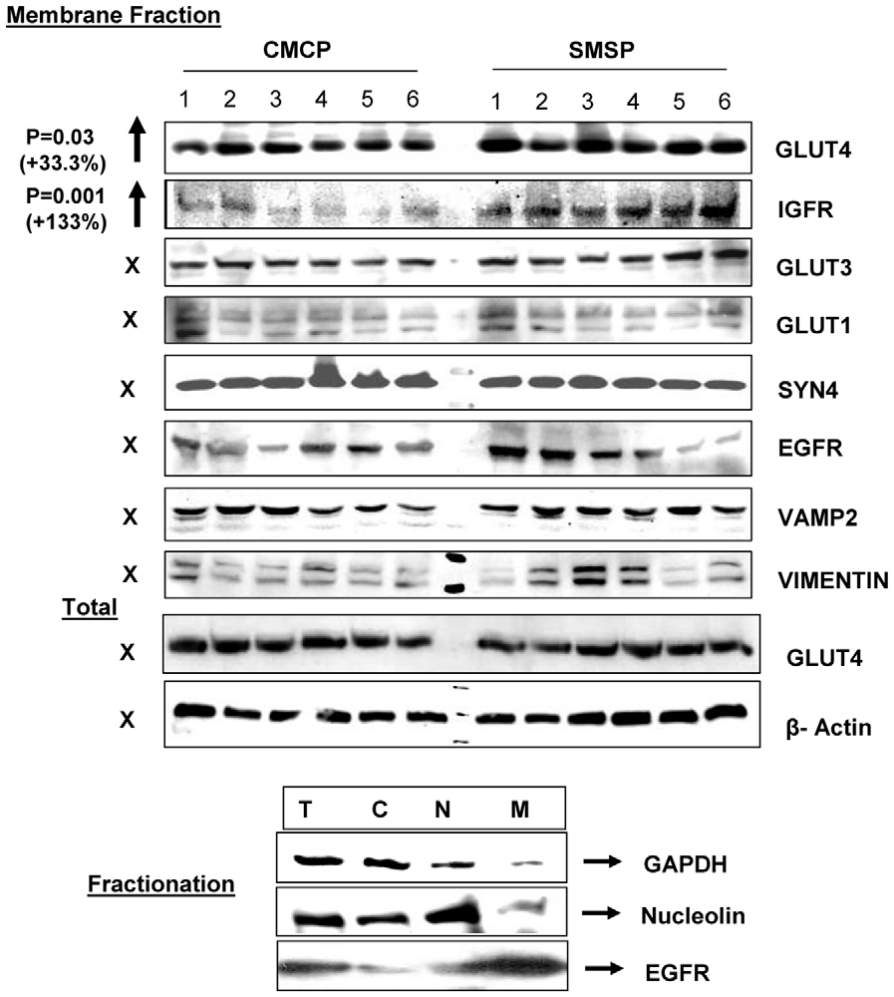
Cellular fractionation of possible target proteins based on microRNA bioinformatics analysis and the insulin-signaling proteins for glucose transport: Membrane fraction. The biochemical fractionations of the membrane proteins were made using Chemicon/Millipore's compartmental sub-cellular analysis technology. Cytosolic, membrane, nuclear and cytoskeleton fractions have been made from SKM tissues from both control (CMCP) and experimental (SMSP) male animals. SDS-PAGE followed by Western Blot analysis was made using 50 µg of total protein from every fractions based on Bradford assay. The localization of GLUT4 and IGFR in IPGR male SKM is significantly increased in membrane fractions when the total GLUT4 protein level remains unaltered in male SKM. GLUT1, 3 and 4 are Slc2 group of glucose transport proteins, IGFR is Insulin Growth Factor Receptor protein, EGFR is used as internal control for membrane localized protein. Vamp2 and Vimentin are two essential regulatory protein markers for insulin signaling and cell differentiation. β-Actin was used as internal control for total protein. **The bottom panel** represents the verification data for the cell fractionation. GAPDH, Nucleolin and EGFR were used for cytosolic (C), nuclear (N) and membrane (M) localized proteins. T represents total protein.

**Figure 6:**
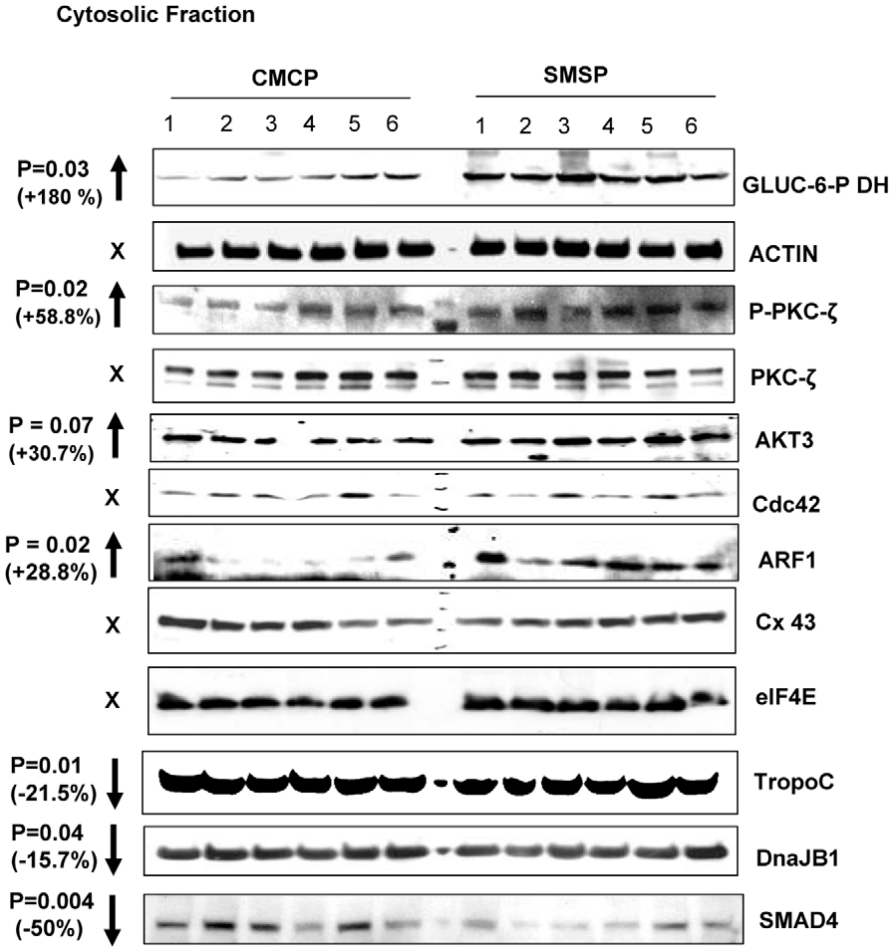
Cellular fractionation of possible target proteins: Cytosolic fraction. Insulin signaling marker is up-regulated along with the metabolic marker glucose-6-phospahe dehydrogenase and ARF1 GTPase, whereas SKM marker Tropomyocin C is down-regulated along with two essential signaling candidates SMAD4 and DnaJ-B1.

**Figure 7:**
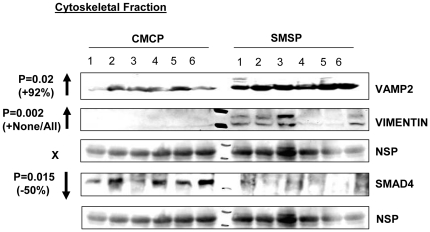
Cellular fractionation of possible target proteins: Cytoskeletal fraction. Insulin-signaling protein VAMP2 is upregulated along with the regulatory protein Vimentin, whereas SMAD4 TGF-β co-SMAD is down-regulated. NSP represents non-specific protein serving as internal control.

**Figure 8:**
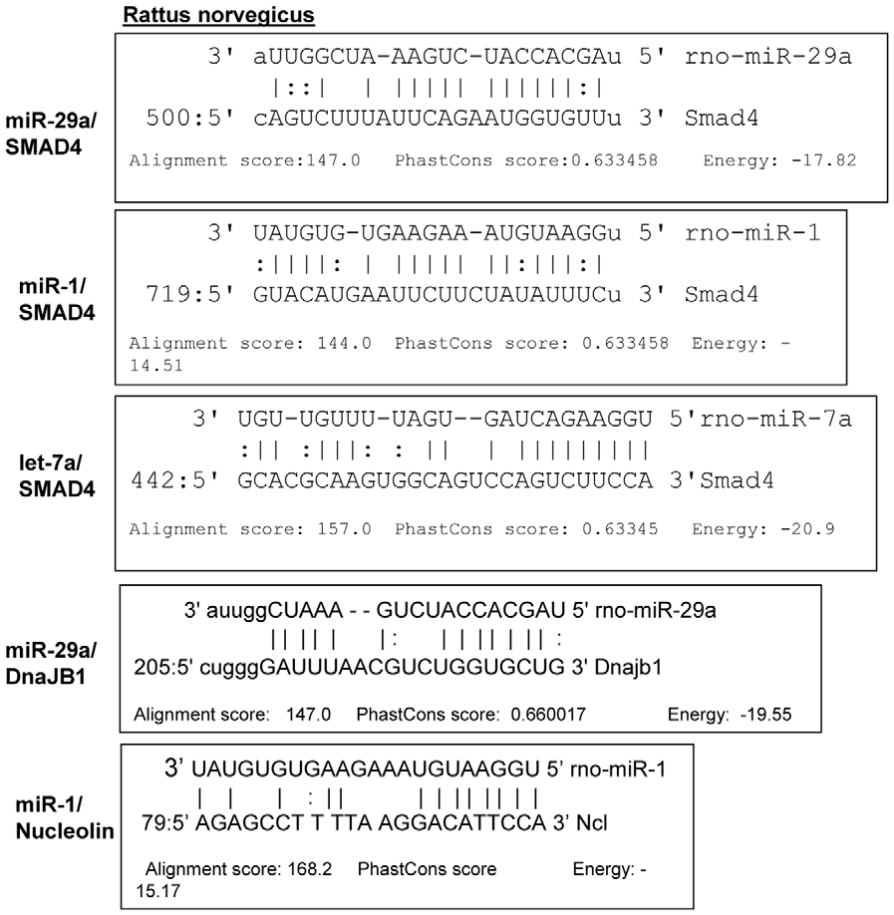
SMAD4, is one of the targets for miR-29a and/or miR-23a: Bioinformatics analysis. miR-1, miR-29a and miR-23a for SMAD4, DnaJ-B1 and nucleolin 3′UTRs. MIRANDA and Sloan-Kettering MicroRNA target analysis site were used for this analysis. Alignment score, PhastCons score and Energy values were cited for all the interactions.

Although LNA based-miRCURY microarray produced results reproducible in further analyzing differences in *in vitro* confirmation of the over-expression of let7a, miR1, miR-29a and miR-23a in SMSP samples, the approach taken here was focused on the identification of target protein genes for the respective microRNAs that affect the glucose transport pathways in SKM. As a first step of this process, bioinformatics tools have been explored; specifically the Sloan-Kettering site for microRNA target identification software was available in open access Internet search. First, we have identified several putative target mRNA 3′UTR sites matching a profound homology in terms of physicochemical stabilities with the microRNA species over-expressed in our system. I focused on those protein factors that were significantly down-regulated as possible direct targets of respective microRNA(s) that regulate or putative regulator of glucose transport process. This approach may significantly reduce the time required for global experimental searches, such as proteomics. The sk. muscle individual samples from six different rats were homogenized and fractionated into four different major fractions in order to see the compartmental localizations of the putative protein factors and their relative levels in control (CMCP) vs. experimental (SMSP) samples. In order to do this experiment, Chemicon International's proven standardized, reproducible, sequential compartmental protein isolation kit was used for dissecting semi-quantitative separation of individual proteins of interest [47], [48]. The cross-contamination of the four fractions was checked (Fig. 5) for the standard proteins. The bottom panel of Fig. 5 shows a little contamination of cytoplasmic protein GAPDH in the nuclear fraction which is probably a part of the perinuclear membrane. EGFR, a membrane-localized protein mostly came into the membrane fraction except little as nuclear fraction, again may be, as a part of perinuclear membrane. Nuclear protein Nucleolin is mostly found in nucleus and some cytoplasm. Nucleolin has translation function in cytoplasm too. We need to remember that it's not ‘all or none’ in terms of localization and function and equilibrium. The cytoskeletal fraction was not tested for its cross-contamination. SDS-gel electrophoresis followed by Western Blotting technique was performed using the extracted cellular proteins sequentially isolated from cytosolic, nuclear, membrane and cytoskeletal fractions. Based on previous knowledge of sub-cellular protein localization, the semi-quantitative estimation of the level of respective proteins by Image J analysis of the band intensities was compared between the two groups. The pattern of expression of protein signaling factors for GLUT4 translocation to the membrane in response to insulin binding to its receptors would tell us the status of the glucose transport activity in the starved (SMSP) muscle samples. GLUT4 expression was found to be increased in the membrane fraction by 33.3% (p<0.05) (Fig. 5) in contrast to the female SKM where there was a similar decrease of GLUT4 expression, both in mRNA and Protein levels [32]. No differences were observed in the total amount of GLUT4 levels (Fig. 5) between the two extreme groups, reinforcing the idea that no transcriptional regulation may be associated with the Glut4 expression in male SKM (Fig. 5). As there was no change of mRNA level between these two groups of male-specific muscle tissues (Fig. 1), it is suggested that the chronic starved male sk. muscle in IPGR rat model behaves differently than that of female counterpart where the transcriptional repression event is demonstrated. Interestingly, Insulin-like Growth Factor-1 receptor (IGF1-R) is also over-expressed in the experimental membrane fraction by 133% considering CMCP as 100%, along with the GLUT4 receptor, probably because SMSP starved cells need more IGF signaling in male sk. muscle under this circumstance for facilitating glucose transport inside the cells. Insulin-dependent and IGF1-dependent glucose transports are two parallel interconnecting mechanisms active in many cell-lines and subcellular tissues [49]–[51]. Importantly, the expression levels of GLUT1 and GLUT3 isoforms of glucose transporter did not change in the membrane fraction at all, implying that GLUT4 is recognized as a major isoform functionally important both in adult sk. muscle and in cultured myocytes-myotubes in both insulin and IGF1-dependent glucose transport activity. As a control, membrane-dwelling epidermal growth factor receptor (EGFR) expression was tracked from two groups as it is known that this receptor functions as EGF-induced membrane glucose translocation in transgenic 3T3-L1 adipocytes [52], and this protein appears to be a bonafied membrane-spanning internal control protein. There was no EGFR expression difference between the groups. Glucose-6-phosphate dehydrogenase (G6PD) is the rate-limiting enzyme in the pentose phosphate pathway. This rate-limiting enzyme is also known to be induced by insulin-dependent activation of phosphatidylinositol 3-kinase (PI-3 Kinase) [53]. The observation that G6PD is highly up-regulated in SMSP muscles (∼180%) suggests a hypothesis that a similar mechanism may exist in this regard (Fig. 6). So, it is expected that GLUT4-mediated glucose transport activity may be switched on through either insulin or IGF1 mediated activation of PI-3K signaling in these SKM samples. Evidence has firmly established the involvement of PKC-ζ in GLUT4 translocation in a variety of adipose and muscle cell lines. The PI3K downstream activator PIP_3_ is a suitable candidate for PKC-ζ activation [54]. This unusual protein kinase C has been shown to bind with Rac1 GTPase (Cdc42) and mediates ‘Actin-remodeling’ in L6 myocytes, leading to insulin-induced glucose transport [55]. In male rat IPGR muscles, PKC-ζ phosphorylation (Thr-410) is activated by 58.8% compared to the total forms of PKC-ζ which remain unaltered (Fig. 6). A similar activation of the PKC-ζ (Thr410 P) was documented in the female counterpart SMSP samples (IPGR) in absence of *ex-vivo* addition of insulin [38]. In this paper, the authors described the aberration of insulin signaling in the food-restricted model of IUGR female rat. In the male counterpart of IPGR sk. muscle, Cdc42 GTPase and β-Actin expression patterns, however, were not changed significantly (Fig. 6) between the groups, suggesting that remodeling may only exist under the experimental conditions and further experiments are required to draw a valid conclusion. ARF1 GTPase is up-regulated by 28.8% in SMSP SKM, suggesting its possible involvement in structural muscle rearrangement for *in vivo* muscle development in IPGR offspring (Fig. 6). Upstream AKT3/PKB expression is also increased by 30.7% in the SMSP groups, but happened to be statistically insignificant (p = 0.07) (Fig. 6). Finally, Vesicle-Associated Membrane Protein 2 (Vamp2) has been implicated in the stimulation of glucose transport activity in conjunction with PKC-ζ. Activation of PKC-ζ induces serine phosphorylation of Vamp2 in the GLUT4 compartment upon insulin treatment in primary cultures of rat skeletal muscles [56]. A ninety-two percent increase of Vamp2 (p<0.05) was found in the cytoskeletal fraction of the restricted group compared to the control muscle, but no change in total Vamp2 protein levels has been observed in the membrane fraction (Fig. 5, 7). Serine phosphorylation of Vamp2 was also increased in the SMSP group as was the total protein in the cytoskeletal fraction of the cell extracts (data not shown). Another interesting observation was the drastic over-expression of a mesenchymal protein marker, Vimentin, in the cytoskeletal fractions of the experimental groups (abruptly increased, p<0.1, Fig. 7) which was initially planned to be included as CS resident marker. This marker protein is usually found in regenerating skeletal muscles in the early myoblast phase of proliferation in the fetus SKM and then found in decreasing levels while in the late stage of differentiation forming myocyte-myotubes particularly in sarcomere development [57]. This is perhaps highly suggestive that the SMSP group of rat SKM under the chronic starved condition creates a more regenerative and mobile Vimentin-expressingmuscle cells for repair and development of new muscle cells from stem cells. Tropomyosin C level was much less in this food-restricted muscle group (21.5% decrease in cytosolic fraction and p = 0.01; Fig. 5) compare to myosin level (remains unaltered, data not shown) suggesting a possible functional significance thriving for regular muscle contractions, probably by exposing more G-Actins for interactions with Myosin motor heads [58]. Most of the protein factors that I discussed and presented here are based on the computer simulation in microRNA binding sites of interest in those putative genes 3′UTR. But, if there is a direct binding of the microRNA(s) that were over-expressed in SMSP sk. muscle with some of the putative targets, we should expect the decrease in protein expression level in the semi-calorie food restriction group. Nuclear protein Nucleolin binds to target 3′UTR for mRNA stability under stress conditions e.g., anti-apoptotic factors [59]. Ncl mRNA have a miR-1 binding site (Alignment score 168.2 and energy for binding mRNA target sequence, −15.17; Fig. 8). The expression of this protein is decreased significantly in SMSP rat muscles by 25% (p<0.05) suggesting that it may play a role in destabilization of protein factors in stressed muscles. Another regulatory protein dnaJ-B1, an isoform of HSP40 chaperone, has the binding site for miR-29a of alignment score 147 and stability −19.55 (Fig. 8). At present, a function for these two proteins associated with muscle development and growth, including glucose transport function, is not characterized. DnaJ-B1 level is found to be significantly (p<0.5) less (15.7% decrease) in the experimental animals compared to the controls (Fig. 6). Gene expression profiling of human sk. muscle suggested dnaJ-B2 is likely involved in muscle growth, remodeling and in stress regulation [60]. More direct questions to be asked in finding out the role of these proteins in the functional aspects of sk. muscle metabolisms and the associated regulations in glucose transport activity. More significantly, another extremely important regulatory protein factor, SMAD4, which is pivotal in TGF-β signaling of muscle growth and differentiation, was found. Central co-SMAD, Mothers Against Decapentaplegic Homolog 4, bears putative binding sites for three different microRNAs miR-1, miR-29a and let7a (Fig. 8). It is tempting to speculate that this particular regulatory protein may play an important role in glucose metabolism either directly or indirectly if proven to be real target. TGF-β-SMAD pathway controls the sk. muscle growth and differentiation under various circumstances and proved to be essential for muscle function both in embryonic and adult mammalian tissues. Muscle-specific conditional inactivation of TGF-β super-family gene, myostatin, modulates the sk. muscle mass [61] and similar gene, GDF8 is known as inhibitory to the muscle growth in mice [62]. Schabort et al [63] demonstrated the role of TGF-β in promoting proliferation and delay in differentiation of mice-origin C2C12 myoblast cells. It is not surprising that SMAD4, a central regulator of TGF-β function in sk. muscle glucose metabolism.

### Transient transfections of precursor micro-RNA(s) and anti-miR(s) in rat L6 skeletal muscle cell-line demonstrate a stimulatory role for miR-29a in glucose transport activity in general whereas miR-23a for insulin-dependent function in the same

**Figure 9:**
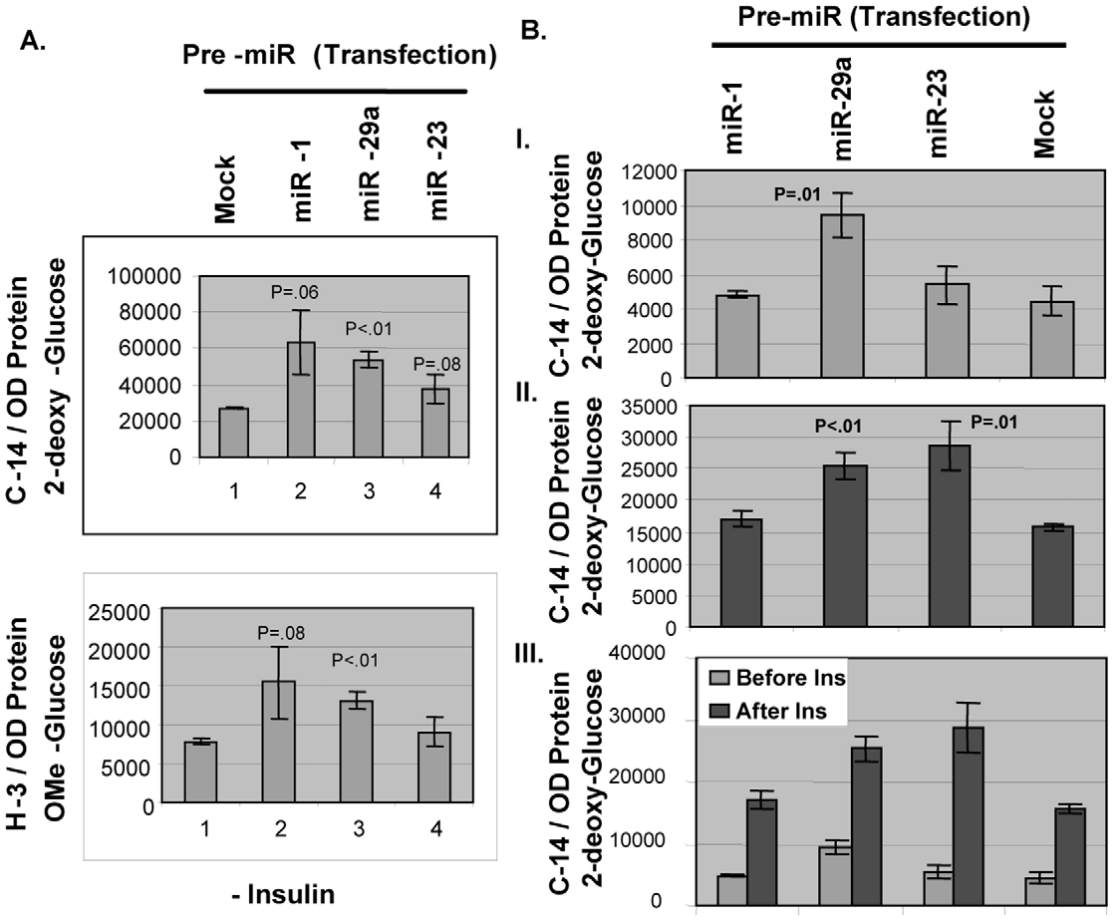
Mimic microRNAs miR-29a and miR-23 up-regulate the glucose transport activities in L6 SKM cell-line as insulin-independent and dependent manner respectively. A. **Pre-miR transient transfection.** 25 nM Pre-miRs were transfected in rat L6 cells according to the protocol as described in method section. ^14^C-labelled 2-deoxyglucose and ^3^H-labelled OMe-glucose were used as substrate along with the non-radioactive substrate (0.15 mM) for glucose transport activity as reversible and irreversible manner respectively. Cytochalasin B is not being added. B. **Pre-miR transfection with Insulin.** Same transfection experiments were done with cytochalasin B (10 µM) added in the transport reaction in presence and absence of 50 nM insulin. Glucose transport assay was performed just (I) before and (II) after adding insulin. (III) Panel was drawn to see the comparison before and after the insulin addition for each mimic precursor microRNA with respect to the control transport of ^14^C-labelled 2-deoxyglucose.

**Figure 10:**
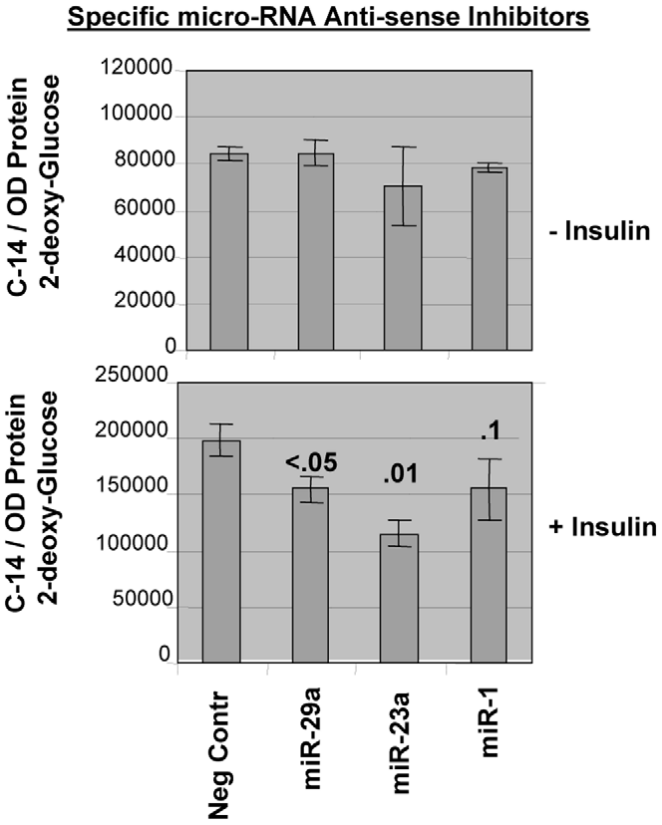
MicroRNA anti-sense inhibitors (miR-29a and miR-23a) down-regulate the glucose transport activity in insulin-added condition in L6 cell-line. Ambion-tested specific endogenous microRNA inhibitors (25 nM) were used in this experiment basically the same way as mimic transfection experiment (A & B). Only, the standard radioactivity amount was increased to 7.5 µCi per 12 well plate for both (I) without insulin and (II) with insulin conditions. Cytochalasin B (10 µM) was added in the transport reactions.

I asked the question directly in an established rat cell-line whether miR-1, miR-23a or miR-29a play a role in regulating glucose transport activity (GTA) *in vitro*, delineating the possible functional correlation of these microRNA(s) over-expressed in SMSP muscle tissues. Although let7a microRNA is found to be a highly prominent potential candidate for this GTA activity in our system, this regulatory RNA molecule proved to be complicated in its function of regulating GTA (data not shown) and thus I have decided to omit let7a data in this publication until further investigation. Ambion Inc. has developed a transfection-based functional assay using synthetic precursor small dsRNA molecules (Pre-miR) that mimic the dose-dependent up-regulation of mature microRNA suitable for gain-of function studies using C^14^-2-deoxyglucose and the non-metabolized glucose analog 3-*O*-(methyl-^3^H)-D-glucose either by dual labeling or individually in presence or absence of cytochalasin B, an inhibitor of glucose transporter-mediated glucose uptake. Pre-miR transfection experiments were performed in L6 myocyte-myotube cells according to the protocol described in Method. Radioactive-glucose uptake inside the cells was compared among Mock vs. Pre-miR transfected cells per OD protein minus cytochalasinB treated (10 µM) transporters-independent transport if added. Fig. 9A shows a picture of total GTA, independent of insulin and cytochalasin B treatment. All of the Pre-miR-transfected cells demonstrate the up-regulation of glucose transport activity both in normal and non-metabolized radioactive glucose substrates added together in the master-mix. MiR-29a significantly up-regulates GTA (p<0.01), whereas both miR-1 and miR-23 tend to increase the transport activity (for miR-1 p = 0.06 and 0.08; for miR-23a p = 0.08 and no difference; Fig. 9A, B) compare to the mock treatment. These results suggested an inhibitory function of some unknown target protein(s) of these microRNA(s) involved in regulating glucose transport uptake in general. The specificity of the glucose transporter-mediated glucose transport process was examined with cells transfected with the same Pre-miRNA(s) under the same condition except that the 50 nM insulin for 30 minutes was added in the culture prior to and during the transport assay. Fig. 9B (I) clearly shows the reduced transport in the presence of cytochalasin B (subtracted the values obtained with cytochalasin B) and recapitulates the results of the previous experiment just before the addition of insulin. After the addition of Insulin, muscle cells demonstrate more than a two-fold induction of total glucose transport, approximately same as other people published elsewhere in L6 cell-line. In this case, exogenous addition of miR-23a resulted in a more than six-fold increase in GTA in response to insulin in comparison to only less than 3-fold stimulation due to insulin addition (Fig. 9B, III). The major active facilitative glucose transporter known in SKM, GLUT4, is insulin-dependent, and is probably responsible for this change of glucose transport activity as also revealed in immunoblot analysis of the skeletal muscle tissues by GLUT4, 1 and 3 antibodies in the membrane fraction (Fig. 5). *In vitro* starved L6 muscle cells may have positively induced the similar microRNA as was in food-restricted animal tissues that will need further experimentation. As the control mock-treated cells had gone through the same treatment and protocol, it is safe to conclude the result in the experimental group. This experiment using the gain of function of mature microRNA(s) incorporated inside the cells, for the first time, reveal two microRNA(s) that up-regulate the insulin-dependent and independent glucose transport processes in starved muscle cells that may explain the over-expression of GLUT4, IGF1-R proteins and other insulin-signaling proteins (e.g., PKC-ζ, Vamp2) in SMSP SKM samples (Fig. 5, 6, 7). Next, it was determined whether I would obtain results in line with the over-expression studies using anti-miR precursor molecules. I knocked out the cellular microRNAs by anti-miR inhibitors developed by Ambion Inc. The ‘ready-to-use’ chemically modified single-stranded RNA molecules bind with target endogenous miRNA specifically depleting their level significantly and has been tested in house successfully for their effectiveness (Ambion Inc.). So, depleting individual miR molecules endogenously by these siRNA-like inhibitors may answer questions about the functionality of these regulatory molecules. While checking the glucose transport activity in L6 myocytes transfected with the anti-miR inhibitors, almost no change was observed in glucose transport activity in the absence of insulin except there was a trend of diminished transport activity for miR-23a of no statistical significance (Fig. 10). But the functional significance of miR-29a, in the absence of insulin, could not be verified under the condition. This suggested that excess miR-29a molecules might have targeted multiple targets, giving rise to functional stimulation of GTA whereas; the endogenous depletion caused unchanged GTA in absence of insulin. However, in the presence of insulin, all the miR inhibitors have effects in reducing the GTA, particularly in case of miR-23a that brought about a statistically significant decrease of glucose transport (Fig. 10; p = 0.01). Anti-miR-29a also showed a significant decrease in GTA (p<0.05), indicating a role for the regulation in glucose transport process in SKM. This was in line with the over-expression experiment in presence of the insulin where there was a significant increase of GTA (Fig. 9B, II) compared to the negative control treated cells (p<0.01). Given the specificity of the LNA-based anti-miR(s), it is not conceivable to imagine that anti-miR-29a would react with other related miR(s) to make the GTA unchanged under the condition of insulin-independent manner. So, considering the gain of function experiment using Pre-miR-29a, it is very likely that the microRNA concentration is critical in targeting multiple factors to affect insulin-independent glucose transport activity. In adipocyte cell-line 3T3-L1, it has been shown by Aibin H et al. [64] that miR-29a/b/c over-expressions through Adenovirus-mediated delivery were accompanied by the inhibition of insulin-dependent glucose transport activity. In our system, the exogenous addition of micro-RNA 29a in rat muscle cells did not alter the effective insulin-dependent total glucose transport as compared to the control mock transfection with or without insulin (Fig. 9B. III). The microRNA miR-1 did not show any effect *in vitro* by changing the GTA either in presence or absence of insulin. Again, presumably, the level of microRNA and its expression in cells seems to be very important in selecting single or multiple targets which are responsible for GTA inside the cells. Further studies are needed using the controlled expression system of microRNAs in order to understand the intricacy of these small RNA elements for regulating sk. muscle glucose transport processes.

### The protein factors SMAD4, DnaJ-B1, and nucleolin, are possible targets of miR-29a, miR-23a and miR-1

**Figure 11:**
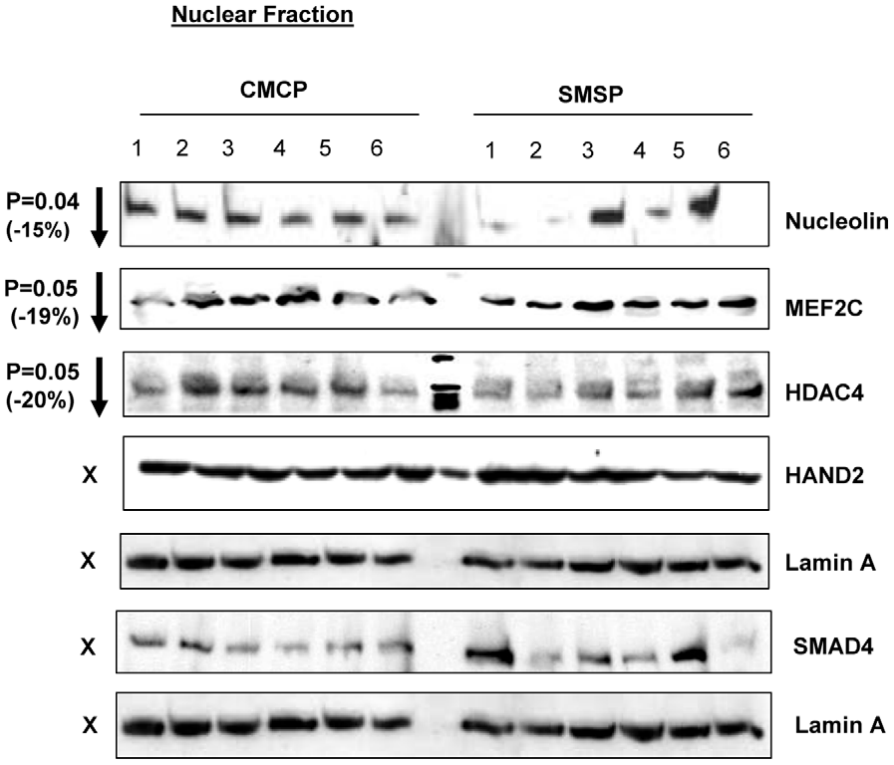
Cellular fractionation of possible target proteins: Nuclear fraction. Nucleolin, a nuclear regulatory factor and HDAC4 and MEF2C, the transcription factors, are down-regulated, whereas HAND2 and SMAD4 remain unaltered. Lamin A served as internal control.

Based on the expression patterns of the various protein factors that have putative target sites in their 3′UTR against the microRNA(s) that were over-expressed in the male SKM of the SMSP group of IPGR rat starvation model, SMAD4, DnaJ-B1 and nucleolin are the prominent ones that may affect the glucose transport activity in skeletal muscles. All of these plausible target protein levels were gone down significantly (p<0.05) in the sk. muscle SMSP samples (Fig. 6, 11). Cytosolic and cytoskeletal SMAD4 levels had gone down drastically in SMSP (by ∼50%) of P values (T-test) 0.004 and 0.015 respectively (Fig. 6, 7), but the nuclear level of SMAD4 remained almost unaltered (14.3% decrease without significance, Fig. 11). SMAD4 is a critical TGF-β pathway co-SMAD that binds to other SMADs (e.g., SMAD3 and SMAD1) for nuclear translocations for transcriptional activation in this pathway. The 3′UTR of SMAD4 harbor the recognition sites for miR-1, miR-29a and let-7a according to the bioinformatics analysis (Sloan-Kettering Institute micro-RNA site, http://www.microrna.org/ microrna, Fig. 8). According to the alignment scores and energy levels, let7a>miR-29a>miR-1 towards SMAD4 target sites (Fig. 8). Unfortunately, let-7a interaction with SMAD4 was intentionally not studied *in vitro* because of the complex nature of this microRNA towards the glucose transport functional assay in L6 skeletal muscle cells (data not shown). DnaJ B1 and nucleolin 3′UTR recognizes miR-29a and miR-1 respectively with very strong affinities (Fig. 8) according to miRANDA alignment score and energy of stabilizations. These protein levels (DnaJ B1 & nucleolin) were found to be down-regulated in the IPGR experimental animals compare to the controls (15.7% and 15% decrease respectively with statistical significance P = 0.04; Fig. 6, 11). Nuclear proteins HDAC4, MEF2C and HAND2 transcription factors were implicated for GLUT4 metabolism in sk. muscle [32], [65], [66] and found to be targeted by miR-1 and miR-23a in bioinformatics analysis. Interestingly, HDAC4 and MEF2C were also down-regulated by 20 and 19% respectively with p values 0.05, whereas HAND2 came out to be unaltered (Fig. 11). I did not pursue HDAC4 and MEF2C because of no GLUT4 transcriptional differences exist between the groups.

**Figure 12:**
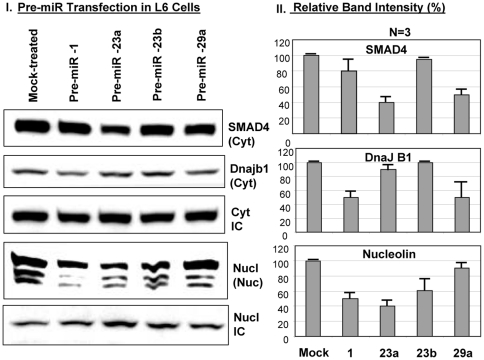
SMAD4 is one of the targets for miR-29a and/or miR-23a: Analysis in Cell-line. PremiR transfections (25 nM) of rat L6 myoblast-myocytes were done and the cell-extracts were subjected to Western Blot analysis for SMAD4, nucleolin and DnaJ-B1 level after 48 hours (I). The triplicate experiments were run in SDS-PAGE/Western Blot to have statistical significant data (T-test) and expressed as relative intensities (%) (I).

**Figure 13:**
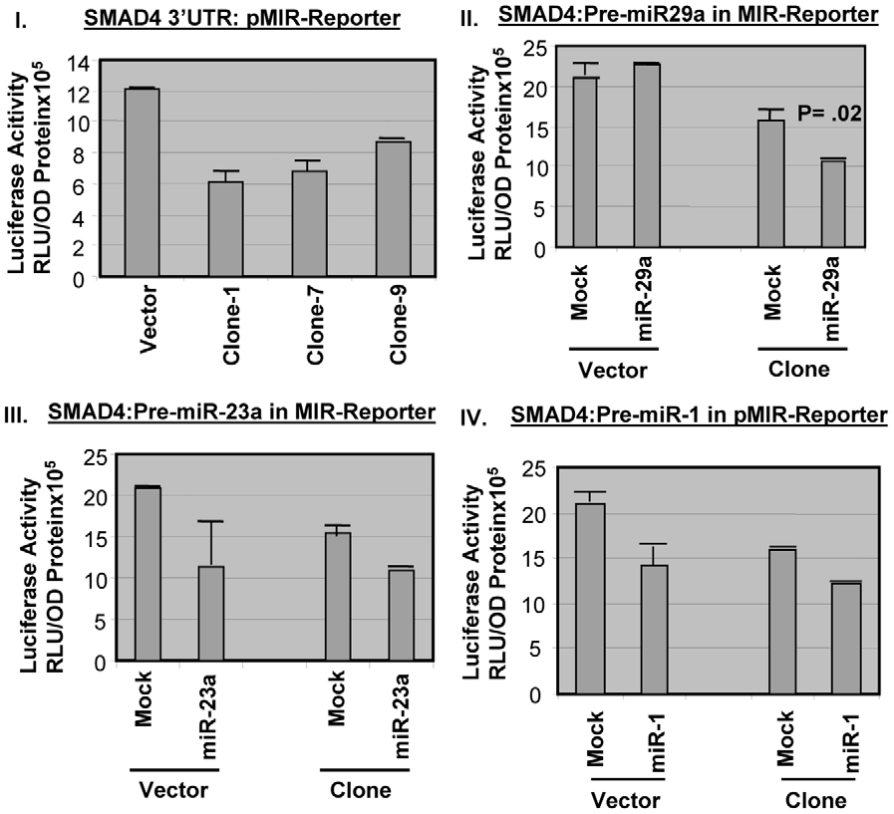
Reporter analysis for microRNA targeting SMAD4 3′UTR. The 3′UTR was cloned in luciferase based pMIR-reporter of Ambion for reporter analysis of microRNA binding sites in its UTR. Plasmid was transfected in presence and absence of precursor microRNAs; 29a, 1 and 23a mimic (25 nM). (I) 3 clones were transfected separately w.r.t. vector without SMAD4 3′UTR. (II) Pre-miR-29a, (III) Pre-miR-23a and (IV) Pre-miR-1 precursors were transfected along with either SMAD4-3′UTR clone or empty vector. Luciferase light units (RLU) were measured in luminometer and expressed as per OD(595) protein measured by Bradford. Triplicate was assayed and expressed as statistical significance by T-test.

Out of three promising proteins factors, SMAD4 has the best chance to be a good candidate because of the severity of the effects observed in animal experiment. In order to check out the target protein's candidacy as a possible target of these microRNAs, the L6 myoblast cells were transfected with the Pre-miR(s) followed by Western Blot analysis to determine the level of the target proteins compare to the mock treated cells. The miR-23a and miR-29a transfections led to the 50% down-regulation of SMAD4 level in L6 cells although there was no miR-23a recognition site for the SMAD4 3′UTR (Fig. 8, 12, I&II). The miR-29a recapitulates the *in vivo* data where there is a drastic diminution of SMAD4 level in rat experimental tissues. The miR-1 and miR-29a target DnaJ-B1 whereas nucleolin is targeted by miR-23 and miR-1 with a statistical significance (Fig. 12, I&II). We need to be cautious about the Pre-miR transfection data where there may be an exogenous binding pressure of the microRNA towards the target. So it is very important to pursue another reporter-based *in vitro* experiment to verify the specificity of the Pre-miR transfection experiment. The Ambion luciferase-based reporter plasmid (miReport) was used in cloning the 3′UTR of SMAD4 (best candidate) in the MCS of pMIR-Report plasmid where the translation level of the luciferase reporter is manipulated by the 3′UTR of the target genes. Then the reporter-based co-transfection was performed using the dual transfections of LNA-based precursor micro-RNA (25 nM) and reporter plasmid (2 µg) in 6 well plates harboring L6 myocyte in triplicates. Fig. 13(I) show the significant decrease of the luciferase activities for clone-1, 7 and 9, which vary from 40%-50% of the original vector. This suggests a strong possibility that the cellular endogenous microRNA(s) may interact with the 3′UTR of SMAD4 to repress luciferase expression, given that there is no transcriptional cross-talk between SMAD4 3′UTR and its upstream promoter elements. Then the transfections of precursor miRNA(s) were performed, along with the plasmid reporter with or without the 3′UTR of SMAD4 gene. The vector controls were retained as a negative control where the administration of microRNA inside the cells might bring about luciferase expression changes without the involvement of the SMAD4 3′UTR interactions. Fig. 13(II) shows the exogenous miR-29a does not alter the vector luciferase expression upon transfection, but represses significantly (p = 0.02) the SMAD4 3′UTR mediated luciferase expression. Whereas, miR-1 and miR-23a transfections results in inconclusively because of the effect of microRNA on the pMIR-Reporter expression alone. But again, the inclusion of the ‘vector alone’ negative control could be erroneous because of the disparity of the neighboring DNA sequences between the reporter alone and its SMAD4 3′UTR construct. It becomes indecisive of the mode of action of miR-23a either to be acting as a direct mediator of gene repression of SMAD4 or targets another protein mRNA factor(s) that regulate SMAD4 expression. In either way, mir-23a has a significant effect on SMAD4 gene expression and has a profound role in insulin-dependent glucose transport process (Fig. 9, 10, 12). The miR-29a is proven to be the real candidate to interact with the SMAD4 3′UTR and also regulate the glucose transport activity in L6 cells. Huang S. et al., [67] reported a link between miR23a and the TGF-β pathway. Stimulation by TGF-β in Huh7 cells up-regulates miRNA cluster ‘miR-23a∼27a∼24’ and this up-regulation attenuates the TGF-β pathway. The 3′UTR of these mRNAs harbor the miRNA target sites (DnaJ1 contains miR-29a and Nucleolin (C23) contains miR-1 sites) and this data is supported by the transfections of Pre-miR(s) in L6 myoblast cells (Fig. 12). MiR-1 targets both DnaJ-B1& Nucleolin; miR-23a target nucleolin & SMAD4 and miR-29a targets DnaJ-B1 & SMAD4 3′UTR. So, miR-29a and miR-23a may directly or indirectly affect SMAD4 expression. SMAD4 protein level in the experimental animals was reduced drastically, particularly in cytosolic and cytoskeletal fractions but not in the nuclear fraction of SMSP samples (Fig. 6, 7, 11) although there was a trend of diminishing value by 14%. This suggested that not much change is expected in the SMAD4-mediated transcription in the male rat model of IPGR, but only expecting the role of SMAD4 in possible perturbation of Insulin signaling regulating Glut4 protein translocation and glucose transport.

### Small inhibitory RNA mediated SMAD4 depletion boosted the glucose transport activity in L6 myoblast-myocyte cells: SMAD4 acts as a negative regulatory protein in sk. muscle glucose transport pathway

**Figure 14:**
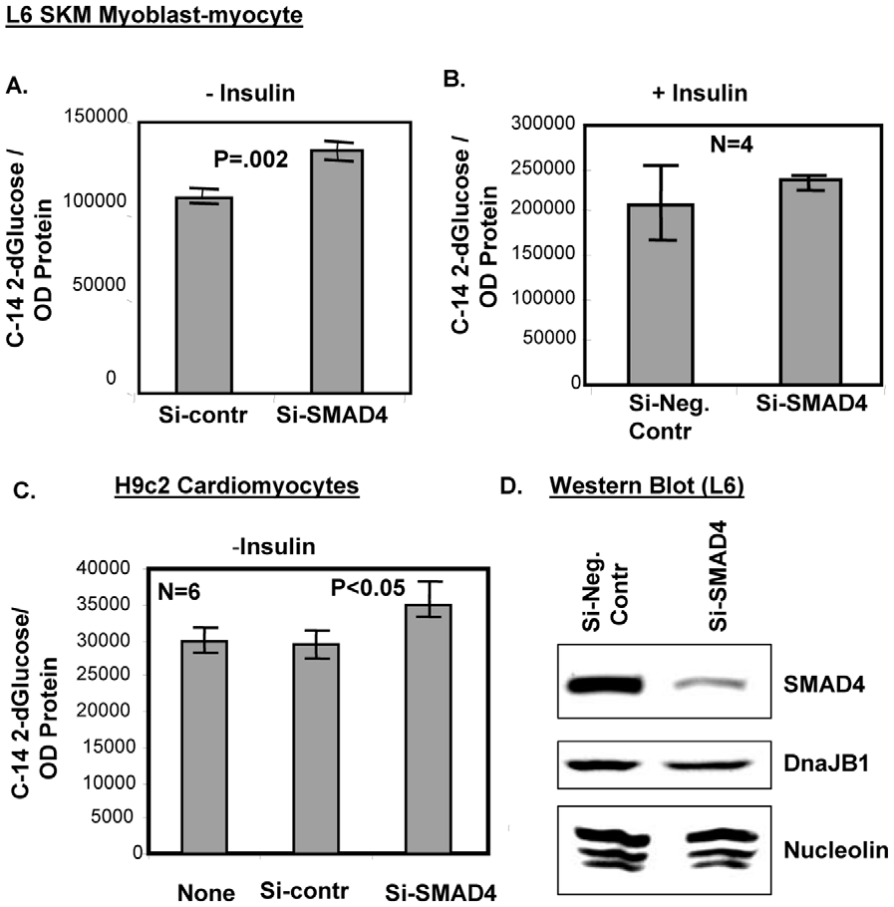
Depletion of SMAD4 activates the glucose transport activity in both skeletal muscle and cardiomyocyte cells. Small inhibitory RNA (25 nM) was used to deplete SMAD4 in L6 cell culture system prior to glucose transport assay using ^14^C-2-deoxyglucose as described in method. Both skeletal muscle cells (I & II) and cardiomyocytes cells (III) were used for this experiment. The transport assay condition is maintained in the same way as was done for Fig. 3. (IV) The Western Blot analysis in L6 cell-lines to show the specificity of siRNA against SMAD4. Dharmacon-designed SMARTpool siRNAs (4 sets) specific to SMAD4 and non-targeting negative control siRNA were used for this experiment according to the manufacturer's instruction.

There are examples where the TGF-β pathway is intrigued for either miRNA biogenesis including maturation and/or specific miRNAs target the TGF-β/BMP signaling proteins for biological activities. The regulation of micro-RNA biogenesis by SMAD proteins is critical for the vascular smooth muscle cell phenotype by TGF-β signaling pathways. A novel role of SMAD in processing the nuclear primary micro-RNA is discussed in the review of Hata and Davis [68]. It has been reported by other investigators that SMAD4 has been targeted by many microRNAs, e.g. miR-224 for cell proliferation and function through TGF-β pathway. Huang S. et al., 2008 [65] published that the microRNA cluster miR-23a∼miR-27a∼miR-24 decreases TGF-β induced tumor suppressive activities in human liver cancer cells (HCC). In another paper, TGF-β reduced the level of miR-29a and vice versa. The inhibition of TGF-β by imatinib restores the level of miR-29a in fibroblast cells [69]. All of these examples signify that the SMAD-dependent TGF-β pathway is a key target of micro-RNA function for various broad-ranged biological activities mediated by miR-23a & 29a. MiR-29a and miR-23a target SMAD4 to regulate glucose transport in rat skeletal muscle. It is very important to ask the involvement of SMAD4, in the glucose transport process in cell culture system. I knock-down the SMAD4 by small inhibitory RNA w.r.t control where the scrambled siRNA is used (Fig. 14D). Two different cell types were used, L6 SKM myoblasts and H9c2 cardiomyocytes for glucose transport activity. The microRNA(s) 29a and 23a, acting directly and indirectly respectively on SMAD4 repressing its glucose transport activities. So, SMAD4 acts like a negative regulator of glucose transport activity in skeletal muscle. Upon depletion of SMAD4, at least partially, an increase would be expected in glucose transport in SKM. Both in sk. muscle myocytes and cardiomyocytes in Fig. 14A, C respectively, shows the increase in GTA in absence of insulin (p = 0.002 and <0.05) by 28% and 16% respectively. Apparently, this effect may be visualized, although not very drastic, nevertheless, in the metabolic scenario, this 28% increase of glucose transport activity in muscle cells carries high significance in the actual context of metabolic perturbation. In H2c9 cardiomyocytes, the efficiency of glucose transport is much less than that of L6 cell-line. Fig. 14B demonstrates that the GTA becomes normalized to the control cells under the treatment of insulin in absence of SMAD4 protein. This suggests that the addition of insulin in L6 cells either complements the level of SMAD4 transcriptionally or induces the insulin-signaling proteins that are counteracted by the SMAD4-mediated inhibition of glucose transport. Unfortunately, at this point, the extent of glucose transport activity increase in skeletal muscle tissues in IPGR offspring animals has not been determined. The phenotypes of the IPGR animals including the metabolic syndrome, if there is any, are also under investigation. Results from two different cell-types, skeletal and heart muscles, probably suggest a universal mode of action of SMAD4 through the inhibition of target proteins in glucose transport machinery in skeletal muscle. This is the first report that the TGF-β pathway is implicated in the skeletal muscle glucose transport function through a common protein factor, SMAD4 has a tremendous implication on the involvement of growth inhibitory signaling in glucose transport process. As there was no significant changes in the nuclear SMAD4 amount (14.3% trend decrease of p value 0.16), it is very likely that this molecule alters the cytoplasmic signaling affecting GLUT4 translocation. The upstream events of SMAD4 will have to be studied in TGF-β pathway for better clarification and role of this protein for the process. The non-SMAD TGF-β signaling might also be important to affect the glucose translocation process in sk. muscle [70]. SMAD4 may be a very good target of intersection between the cell survival pathway, such as IGF and growth suppressor pathway TGF-β. The growth-inhibitory action of TGF-beta 1 on the IGF-I-induced mitogenic effect has been tested by Mincion G., et al. 2003 [71]. IGF-1 also inhibits the transcriptional responses of TGF-β by suppressing the activation of SMAD3 protein in PI3K/AKT dependent way [72]. In our studies, the SMSP (IPGR) male adults express IGFR significantly (Fig. 5) that might down-regulate the TGF-β pathway in an unknown mechanism. These demonstrate the cross-talk ability of these two opposing signaling pathways. In this semi-calorie starvation model of IPGR, particularly in the male skeletal muscles, the plethora of molecular evidences, both *in vitro* and *in vivo*, strongly suggested a negative role of the TGF-β pathway central protein factor, SMAD4, in the normal physiological function.

### Conclusion

As a first step of elucidating skeletal muscle GLUT4 gene-expression pattern in the IPGR offspring, GLUT4 mRNA went down in the IPGR female offspring by 35% whereas for male, remains unaltered. The sharp decrease in GLUT4 gene expression for female offspring is due to the transcriptional repression because of the HDAC1/4 recruitment aberration on the upstream promoter of GLUT4 (MEF2 and MyoD specified) and subsequent changes in the histone-modifications directed to the less transcription of this gene. On the contrary, it was found that the membrane GLUT4 is localized more in the male IPGR samples (Fig. 5) in contrast to the female. This intrigued me to investigate the other genetic factors that affect the expression status of the sk. muscle. While investigating the epigenetic marker status of the male counterparts, I have decided to explore the microRNA status of the male counterparts that might be targeting the signaling pathways leading to the regulation and fine-tuning of glucose transport activity both under normal physiological and IPGR starved conditions. MiR-29a and 23a turned out to be important for efficient glucose transport activity. Both of them target a very critical signaling factor in the TGF-β pathway, called SMAD4, either directly binding to its 3′UTR or indirectly with other factors respectively. This finding postulates that under normal physiological condition, these microRNAs manipulate SMAD4 expression in the translational level, so that the firing of the glucose transport remains in its basal level in the sk. muscle. Now, on starvation stress, based on the semi-calorie food restriction model in rat, these microRNAs are over-expressed that repressed this co-SMAD's expression, probably transiently, resulting in the activation of GLUT4-mediated glucose transport. There is a possibility of another group of microRNAs which are under-expressed in the experimental SMSP samples (Fig. 2) and those could activate the glucose transport inside the cells, by targeting plausible activators of insulin and/or IGF signaling pathways yet to be determined. So, the systematic isolation of microRNAs in the IPGR skeletal muscles, both in male and female samples, would reveal a number of known and unknown protein factors regulating the influx of glucose in skeletal muscles. The identification and targeting of positive and negative regulatory proteins through microRNAs would reveal the complicated metabolic cross-talks among major cell-signaling pathways, where the glucose transporters demand the central focus of studies.

In summary, we have provided strong evidence of the role and plasticity of specific microRNA to manipulate gene expression leading to an alternation of skeletal muscle glucose transport. The miR-29a and miR-23a are two such micro-RNAs identified that modulate the glucose transport pathway *in vitro* as a general and insulin-dependent manner. A critical target regulatory protein has been identified and appears to function as a negative regulatory element in the glucose transport system. This target protein SMAD4, whose 3′UTR contains the binding sites for miR-29a, let7a and miR-1 that are over-expressed in the IPGR male sk. muscle samples. Overall, this paper opens up a new field of investigation in glucose transport pathway regulated by a group of microRNAs operating under starvation stress or in certain physiological conditions. As expected, under stress conditions, including starvation, aberration of the expression of specific microRNAs could alter the metabolic status of the muscle cells, and may lead to pathological conditions such as metabolic syndromes.
